# A Qualitative Study to Appraise Patients and Family Members Perceptions, Knowledge, and Attitudes towards Venous Thromboembolism Risk

**DOI:** 10.1371/journal.pone.0142070

**Published:** 2015-11-04

**Authors:** Claudie Haxaire, Cécile Tromeur, Francis Couturaud, Christophe Leroyer

**Affiliations:** 1 LABERS, EA 3149, Université Européenne de Bretagne, Université de Brest, Brest. France; 2 CERMES3,Université Paris Descartes, EHESS, CNRS UMR 8211, INSERM U988, Paris, France; 3 Université Européenne de Bretagne, Université de Brest, EA3878, IFR148, Hôpital La Cavale Blanche, Département de Médecine Interne et Pneumologie. Brest, France; IIBB-CSIC-IDIBAPS, SPAIN

## Abstract

**Objective:**

This study aimed to examine perception, knowledge and concerns developed by patients and their family as regards venous thromboembolism (VTE) risk.

**Methods:**

We conducted a qualitative study. Participants were: (1) patients with unprovoked VTE with either factor V Leiden mutation or G20210A prothrombin gene mutation or not; and (2) their first-degree relatives. Interviews took place mostly at Brest University Hospital. Participants produced narratives of the patient’s illness, stressing their perception of the disorder, its mechanisms, etiology, circumstances and risk factors. Interviews were audiotaped and transcribed verbatim. On an ongoing basis, central themes were identified and data from narratives were categorized by these themes.

**Results:**

A total of ten patients and 25 first-degree relatives were interviewed. Analyses of patient’s narratives suggested 4 main themes: (1) concerns about initial symptoms and suspicion of VTE. The longer the duration of the initial phase, the more likely anxiety took place and persisted after diagnosis; (2) underestimation of potential life-threatening episode once being managed in emergency; (3) possible biographical disruption with inability to cope with the event; and (4) secondary prevention attitudes motivated by remains of the episode and favoring general prevention attitudes. Analyses of the first-degree relatives narratives suggested 3 main themes: (1) common interpretation of the VTE episode shared within the family; (2) diverse and sometimes confusing interpretation of the genetic status; and, (3) interpretation of clinical signs linked to VTE transmission within the family.

**Conclusions:**

Construction of the risk of VTE is based on patient’s initial experience and shared within the family. Collection of narratives illustrates the gap between these perceptions and current medical knowledge. These results support the need to collect the perceptions of the VTE episode and its consequences, as a prerequisite to any health education process.

## Introduction

Venous thromboembolism (VTE) is a multifactorial disease caused by hereditary and acquired risk factors [1]. More than twenty years ago, with the discovery of activated protein C deficiency due to a factor V mutation, closely followed by the prothrombin gene mutation, it indeed became clear that VTE hereditary factors are common [2, 3]. Later, we, as others, showed that the risk of thrombosis in first-degree relatives of patients with VTE and factor V Leiden or the prothrombin 20210A gene variant was higher if they had the same abnormality compared to neither abnormality [4–6]. However, in a large cross-sectional study comparing the risk of VTE in first-degree relatives of patients with a first unprovoked VTE, we showed that the presence or the absence of factor V Leiden of the prothrombin G20210A gene variant had little impact on the risk of VTE in their first-degree relative [7]. An even more striking finding was that relatives of younger patients had a much higher risk of thrombosis than relatives of older patients. The main hypothesis was that patients with a first unprovoked VTE at young age and no detectable inherited thrombophilia are likely to have an unknown thrombophilia that have yet to be discovered and that these defects increase the risk of thrombosis in their relatives.

Meanwhile, frequent interactions between patients, their family members and physicians from our team during this study shed light on the complex mechanisms developed by participants to adapt their VTE risk perception and to adopt potential preventive attitudes. It also appears that patients and family members interacted on these topics, especially in the context of the discovery of genetic variations in their family. Keeping in mind the interest for caregivers of a better appraisal of actual patient’s perceptions and knowledge, we herein developed a qualitative approach and worked on situations of patients and their first-degree relatives who had received an announcement on VTE risk factors.

## Methods

This study was nested in a previous study of factors that predicts risk of venous thrombosis in 1,752 first-degree relatives of 369 patients with unprovoked symptomatic venous thromboembolism (VTE), either pulmonary embolism (PE) or deep vein thrombosis (DVT). Patients and first-degree relatives were recruited from 5 centers in Canada and one center in Brest, France. The French center included 158 patients and their first-degree relatives. Data on participants had been previously described [7]. Following the completion of the previous study, results of genetic testing have been mailed to all participants (patients and their first-degree relatives). In the presence of a genetic abnormality, first-degree relatives have been provided with a leaflet summarizing current recommendations as regards VTE risk factors management. In addition, at the time the study was published, a summary of the main findings has been mailed to all participants.

Due to the lack of literature on perception of VTE risk by patients and their relatives, and the exploratory nature of the study, we herein have favored a qualitative study design on the families included in France.

### Conceptual Framework

We proposed to work on situations of patients and their first-degree relatives who had received an announcement on VTE risk factors. Such an announcement potentially induces a state that we studied as *liminal*, rich in existential revisions [8]. To get access to practical knowledge that helped families in managing uncertainty and potential related psychic suffering and in order to grasp the singular experience of illness, we have collected illness narratives from patients themselves and their first-degree relatives [9, 10].

### Sampling

As, in our previous study, unprovoked VTE at a young age was associated with an increased risk of VTE in patients' families, we herein focused on younger patients (7 of 10 under 45 years-old) and their family. For this exploratory research, we aimed at recruiting participants that potentially reflects maximal diversity [11]. Eligible patients had: (1) either the factor V Leiden or the G20210A prothrombin gene mutation abnormality or not; and (2) an episode of symptomatic PE or an episode of symptomatic isolated proximal DVT. Patients’ family had to consist at least of one parent and of one sibling (e.g., first-degree relatives). At least one episode of VTE had to be present in past history of at least one first-degree relative. Patients and their first-degree relatives’ places of residence were preferably located nearby Brest University Hospital, thus facilitating potential adhesion to the study. In the case of a first-degree relative living in another area (France or abroad), attention was paid to offer appointment facilities (e.g., during vacations and legal holidays). We used the concept of data saturation to determine the number of interviews: saturation was assumed when subsequent interviews yield no additional data.

### Data Collection

Participants were interviewed face to face by CH mostly at Brest University hospital outpatient clinic, that is, the same location as in our previous study. In five cases, interviews were performed by CH at the patients’ home. The medical team introduced CH to participants as an anthropologist teaching in medical school and working on therapeutic patient’s education. The interview duration was between 30 and 90 with a mean time of 45 minutes. Trained research assistants transcribed the audio taped interviews verbatim.

As stated above, each patient and his/her first-degree relatives produced a narrative of the episode of the patient's illness, stressing their perception of the disorder, its mechanisms, etiology, circumstances, risk factors, etc. At the end, participants were asked to draw a family chart and to make any relevant comment on the topic.

### Data Analysis

A qualitative interpretive approach was taken, combining thematic analysis with constant comparison [12]. In each narrative, attention was also paid on chronology of themes. Two investigators (CH, CL) carried out the analysis and their results were then compared. On an ongoing basis, central themes were identified and data from narratives were categorized by these themes.

### Ethics Statement

Our Brest University Hospital scientific and ethic board approved the study. A specific written informed consent for participation in the study was obtained for all participants.

## Results

**Fig 1:**
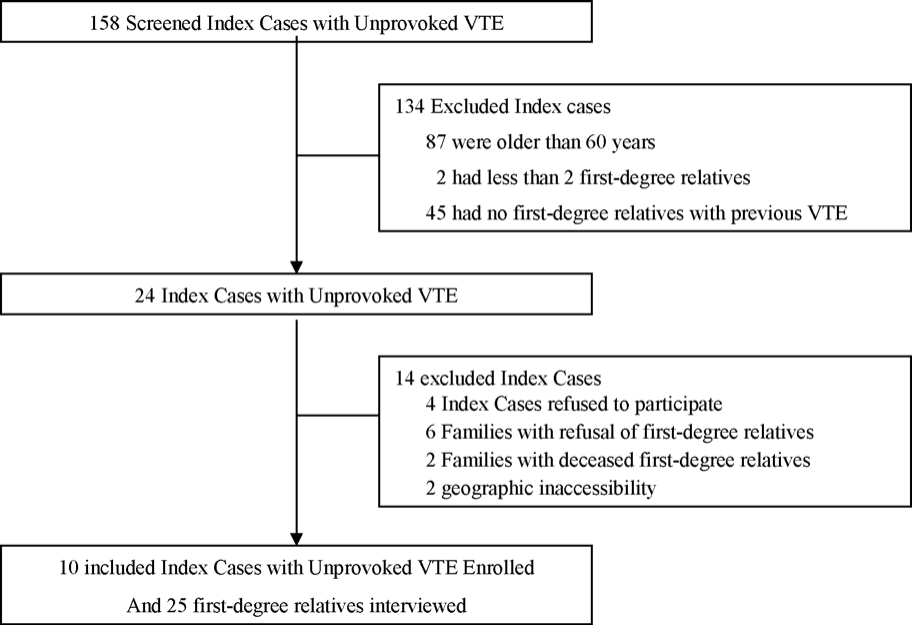
Selection of patients and their first-degree relatives.

Selection of participants is detailed in Fig 1: of 24 potential families initially screened as representing the greatest possible range of variability among the participants, 10 agreed to participate. A total of 10 patients and 25 first-degree family members were interviewed. For another 12 first-degree relatives potentially eligible, we failed to schedule an interview appointment during the study period (remote living area in eight cases, secondary withdrawal of informed consent in four cases). As a consequence, for patient 77 bis, no family member with past history of VTE was interviewed. For patient 92, attempts to scheduled any family appointments failed. Table 1 presents main characteristics of patients and family members. The patients’ sample was unbalanced in terms of sex ratio (eight women, two men) and original VTE manifestation (seven PE, three DVT). In two families (77 and 77 bis), patients were first cousins. Discrepancies between actual genetic status as regards the presence or absence of genetic abnormalities and narrations contents provided by patients or first-degree relatives were found in three families.

**Table 1:** Characteristics of patients and their first-degree relatives.

Patients						First-degree relatives				
Patient identifier	Age [yrs]/ sex	Education level	DVT/PE On anticoagulant at the time of interview?	Genetic mutation identified?	Discordance between genetic status and narration?	Identifier [relationship]	Age	Education level	VTE?	Genetic mutation? / discordance between genetic status and narration?
19	35/F	higher tertiary	PE/ no	no	no	19A [mother]	67	lower tertiary	yes	no/no
						19B [brother]	39	higher tertiary	no	no/no
26	55/F	general elementary	PE/ no	no	no	26A [mother]	84	general elementary	yes	no/no
						26B [father]		general elementary	yes	no/no
						26 C [sister]	54	general elementary	no	no/no
						26D [brother]	50	basic vocational	no	no/no
						26E [brother]	58	intermediate	no	no/no
						26F [daughter]	30	general elementary	no	no/no
						26G [son]	28	basic vocational	no	no/no
27	29/F	lower tertiary	DVT/ no	yes	no	27A [mother]	54	general elementary	yes	no/yes
						27B [father]	57	basic vocational	yes	yes/yes
						27C [sister]	19	student	no	no/yes
56	60/F	basic vocational	PE/ yes	no	no	56A [mother]	80	basic vocational	yes	no/no
57	29/F	higher tertiary	DVT/ no	no	yes	57A [father]		basic vocational	no	none/no
77	24/F	basic vocational	PE/ no	yes	no	77A [mother]	53	general elementary	yes	yes/yes
						77B [father]	55	basic elementary	no	yes/no
						77C [sister]	30	basic vocational	no	yes/no
						77D [sister]	24	basic vocational	no	yes/no
77 bis	25/M	lower tertiary	PE/ no	no	no	77bisA [mother]	50	general elementary	no	no/no
						77bisB [father]	50	basic vocational	no	no/no
92	32/F	higher tertiary	PE/ no	yes	no					
151	58/M	higher tertiary	DVT/ yes	yes	no	151A [mother]	82	lower tertiary	yes	yes/no
						151B [sister]	55	higher tertiary	no	yes/no
152	25/F	lower tertiary	PE/ no	no	no	152 A [mother]	55	lower tertiary	yes	no/no
						152B [father]	55	basic vocational	no	no/no
						152C [sister]	35	Basic vocational	no	no/no

### Patient’s Narratives Initial Symptoms and Suspicion of VTE

The majority of patients started their narration by the initial VTE episode description; the concern of possible genetic transmission to descendants was however put forward by one patient, before any interaction with the interviewer, thus disrupting narrative’s sequences. *"Doctors told me I have the gene …that there is a risk that my family has also the gene*…*finally*, *my mother*, *my sister and my daughter have the gene " (151*, *58 years old male*, *16 years since diagnosis*, *interrupting the interviewer at the very beginning)*.

Descriptions of these initial VTE episodes differed, pending on the nature, PE and/or DVT of the episode. Initial symptoms of DVT, such as leg swallowing or leg pain were easier to identify than initial symptoms of PE. Leg pain was however not always related to a suspected DVT. Patients otherwise may kept in mind a specific kind of pain, previously experienced and described by their relatives as DVT symptoms. In the case of a PE episode, patients may have experienced misleading symptoms. *“I woke-up with a terrible back pain…I paid a visit to my physiotherapist…then pain increased and kept me awake” (152*, *25 years old woman*, *four years since diagnosis)*.

Erratic patients diagnostic work-up may have taken place, not only by misinterpretation of symptoms by patient’s physicians, but also by family members: close relatives who experienced a past VTE episode may or may not help to raise VTE diagnosis, pending on their own past VTE episode symptoms. The longer the duration of this initial phase, the more likely anxiety took place and persisted after diagnosis. *“10 days before a proper diagnosis was made…every one told me I was lucky…afterwards*, *that’s what bothers me a lot” (19*, *35 years old female*, *10 years since diagnosis)*.

### VTE Episode Management

After being admitted to hospital for PE, patients generally expressed their relief and did not immediately felt in danger. Interpretation by patients of close relatives verbal or non-verbal interactions was mentioned as a way to appraise the VTE episode. *“I did not especially felt that my life was in danger*, *but then I saw that my mother was so troubled” (77*, *26 years old female*, *2 years since diagnosis)*. Several patients became aware of the potential seriousness of the PE episode only after proper interaction with physician. “*I believe I realize afterwards*, *as doctors told me I must understand that what happened was a serious matter” (92*, *opus cited)*. In the case of DVT patients whom no PE event had occurred whithin the family, knowledge of possible PE complication resulted from medical team information and contributed to the perception of severity.

### Biographical Disruption

VTE occurrence interfered with daily life and projects in these mostly young patients. VTE episode occurred sometimes so close to a final degree examination that the patient was prevented from attending the session. *“I cried*, *I felt it disgusting” (57*, *21 years old female*, *5 years from diagnosis)*. Both DVT and PE patients may have been unable to cope with the event and its consequences. *“I did not pass my exams*, *I felt unable to concentrate…I was depressed” (19*, *35 years old woman*, *5 years from diagnosis)*. In other cases, a specific goal, such as being successful in the new job, kept patients up to cope with their status and in that instance, preventive measures were felt reassuring. *“Weather was so hot and with my contention stockings (lough) it was a bit difficult*, *but meanwhile I told myself*, *well*, *I felt anxious not to wear those stockings at work” (92*, *32 years old female*, *5 years from diagnosis)*. Patients who expressed difficulties at this time of their life retrospectively claimed that a psychological support, which had not been proposed at that time, might have been helpful *(19*, *35 years old woman*, *opus cited)*.

### Secondary Prevention

Patient’s opinions on anticoagulant therapy (antivitamin K at that time) differed as treatment durations differed. As expected, difficulties in keeping INR ratio within therapeutic range and subsequent blood sampling were mentioned. A patient on long-term anticoagulant treatment expressed the need to experience a treatment interruption to test for its potential interest. *"Well*, *I am on treatment for the rest of my life…it is not so hard…yes I take my pill daily…I stopped one month and then my fourth phlebitis occurred” (151*, *58 years old male*, *16 years since diagnosis)*. In our group of unprovoked DVT or PE patients, especially when a family member had previously experienced a PE event, narratives showed concerns on possible recurrences. Only few participants mentioned abstract knowledge of possible VTE recurrence as a motivation for secondary prevention. In others, the presence of remains was mentioned as a reminder of the interest, both of staying aware of possible VTE recurrence and of secondary prevention measures. Those remains were symptoms for some patients (leg pain, thoracic pain, fatigue…), knowledge of sequels for others. *“The clot is in my thigh…I don’t know from the medical point of view…but I understand that the clot is here and knows me…once it adheres to the vessel wall*, *yes” (57*, *29 years old female*, *3 years since diagnosis)*. Hence, not only symptoms, but also knowledge and interpretation of the nature of infra-clinical sequels served as reminders.

Preventives measures were re-interpreted with emphasis on advices in the event of long travels and on the interest of practicing physical activities. In some patients, regular walking was mentioned as a proxy for contention stockings. *“No*, *I can’t stand stockings*, *they are useless*, *my legs are nice…as I walk 60 kilometers a week…I have friends…she is like me*, *she walks a lot*, *but the husband has a lot of problems…not surprising I told him*, *you have at least 30 kilos exceeding for your height” (151*, *58 years old male*, *16 years since diagnosis)*. Besides promoting the interest of physical activity, other preventive measures applicable at the population level have been endorsed by patients, such as avoidance of smoking and drinking and being on a healthy diet (“five fruits and/or vegetable a day”). Keeping body weight within normal range or fighting against obesity were preventive measures put forward. Spontaneous mention of pathophysiological knowledge was infrequent, mostly made in support of these preventive measures. *“Yes*, *I was told that I don’t exercise*, *I stay home” (26*, *55 years old female*, *16 years since diagnosis)*. This patient endorsed her son’s explanation. “*And I know that she doesn’t drink a lot either… so… it’s not good for blood circulation… so she doesn’t make an effort*, *so for me it’s the same” (26G*, *28 years old son)*.

### Family Member’s Narratives Interpretations Shared within the Family

The relevant signs, and to a certain extent the interpretations of the causes of VTE, were shared within families, whether there is good communication and a close relationship among all the members of a large family (77, six members and 77 bis, three members) or, on the contrary, no communication in an equally large family (26, eight members) and just as close geographically, but in which each person takes care of himself. These interpretations resulted from an analysis of the same context of the initial VTE episode. This is the case for the following family, which attributed VTE episode of the patient to tiredness. “*Because of the fatigue” (77*, *55 years old father)*.*“Well when she is tired*, *yes*, *she can feel that or she has more trouble breathing …when she begins to be tired*, *you can see that then it starts to… since she has the pastry in addition*, *it’s true that always being on your feet” (77*, *26 years old sister)*. Everyone was eager to prevent another episode by making sure this patient doesn’t get overtired, such as her colleagues: “*when I work too many hours*, *they tell me to slow down a little” (77*, *26 years old female*, *2 years since diagnosis)*. When a nephew in the family, who did not take part to the interviews, also developed a PE episode, they attributed it to the same causes, that is, fatigue and stress *(77 bis*, *25 years old male)*. The same interpretation prevailed, even though the members of another family only get together from time to time. The patient, a pre-menopausal women, was seen as depressed, not getting any exercise, smoking, with an unhealthy lifestyle. “*Yes*, *that’s why*, *she doesn’t make any effort at all and… well it’s true that we… compared to us… we exercise” (26*, *28 years old son)*. We observed autonomous adoption of preventive measures that were derived much more from the interpretations of VTE episodes shared within the families than from recommendations prescribed by health care personnel. Whether or not physicians endorsed these autonomous preventive measures was not mentioned.

### Impact of Genetic Status Knowledge within the Family

Although patients and family members received individual results of genetic status, few families developed these findings in the interviews. On the one hand, three fathers interviewed worried about this potential transmission and thus take special care of their child who has a positive test (families 151-57-27). On the other hand, a mother felt reassuring to know what is at stake (family 27). When a whole family shared genetic results, interpretation of genetic transmission leaded to a false reinsurance in one case: it happened that only male carried Factor V Leiden mutation in that family (family 77); the feeling of being protected—as free from the genetic mutation—in descendant of female was questioned as a VTE episode then occurred in one of their child. Families were even more dismayed by the fact that the absence of the gene does not preclude the possibility of an episode. In one family, the fact that some have a detectable genetic abnormality while others do not may be a source of confusion, as for this father: he developed DVT without “*having the gene”* that his wife and daughters *“have”*. Their spontaneous search to find out *“from which side it comes”* was frustrated by the results that did not find “the gene” on the side they expected. *“My wife is the one with the gene and I’m the one who had phlebitis*, *so you see…and apparently they all carry the gene except me–I have nothing and I’m the one with phlebitis” (Father of 27*, *57 years old male*, *21 since diagnosis)*. He connected the genotype with the phenotype at risk according to him: his eldest daughter who developed phlebitis has the at-risk phenotype in his opinion *“Yes… she is big on top of it all*, *so maybe that had something to do with it too”*.

### Signs of VTE Transmission among Families

VTE transmission, from a family perspective, was based on shared interpretation of clinical signs: bad legs and varicose veins, leg swallowing, overweight or absence of exercise “*My mother*, *I think she might have had phlebitis too…with such swollen legs…well we all are overweight” (27*, *29 years old female*, *6 years since diagnosis)*. These signs are linked, from their point of view, to a blood disturbance. Keeping in mind that oral and written information have been previously provided by medical staph, for the majority of participants, distinction between venous and arterial pathological events remained unobvious. In one family, VTE disease and heart attack were attributed to something about the blood. This latter family, which tested negative for the two factors looked for in the study, was concerned about inheriting various blood-related problems, some of which had been tragic, and they incriminated a “gene” as the casual factor, a gene that they did not have in the study tests.

## Discussion

Our qualitative study aimed to appraise patients and family members perceptions, knowledge and attitudes towards VTE risk, in the context of a genetic study of unprovoked episodes. Three main perspectives can be extracted from the narrative’s analyses described above: first, patients and family members are faced with the challenge to appraise the risk of DVT or PE recurrence; second, potential physical and psychological impact of VTE may lead to profound life disturbances; third, preventive measures, a strong concern within the families, may resulted, not only from doctor’s prescriptions, but also from a collective attempt to interpret and then prevent the cause of the VTE episode, using autonomous methods.

### Unprovoked VTE and the Risk of Recurrence

The concern of possible recurrence was expressed in the subgroup of seven PE patients as well as in the three DVT patients. Conversely, initial underestimation of potential severity was apparent from narratives of both PE and DVT patients. As a consequence, our results suggest that this risk perception is not related to VTE site. Instead of that, in some cases, interactions with patient’s physicians and/or family members were necessary to appraise this risk in those patients initially reassured, after hospital admission, by quick management and close follow-up. In those interactions, VTE events and secondary prevention attitudes made sense for patients. Working on narratives gave us access to theses co-constructions of sense. According to Mattingly and Garro, narratives characteristically relate the subjects’ experience of VTE episode and the outside world in which the VTE episode occurred [10]. Narratives help to grasp how the subjects understand and construct the meaning of what happened to them. Kaptein and co-workers have previously studied VTE risk perception in a different setting [13]. The authors applied the Common-Sense model, using Illness perception questionnaire, to a group of more than 170 individuals with a genetic predisposition to VTE, of whom 95 had a past history of VTE. Among the studied variables, a personal history of VTE appeared as a good predictor of VTE risk perception.

### Impact of VTE

VTE, as a potential life-threatening event, has the potential to affect both physical and psychological status. Not surprisingly, our participants reported significant alterations in these domains. Psychological alterations mentioned included both anxiety and depression and occurred within a variable time lag. Noteworthy, a delayed diagnostic process was reported as a cause of anxiety level enhancement. Disruption of life projects, as a consequence of these alterations, was a major concern for our fairly young patients. Depression status was reported as a leading cause that impeded patients from maintaining activities and attaining their goals. However, patients who coped with VTE episode consequences and achieved their life projects mentioned a good adherence to preventive measures.

Measurable global consequences of VTE have received little attention, mainly through study of quality of life. Studies have made use of both generic [14] and/or specific [15, 16] instruments and demonstrated significant alterations. Specific focus on anxiety and depression suggested that PE patients suffered from high levels of depression and anxiety [17]. Whether or not testing for thrombophilia added further alterations of psychological status was unclear in our patient’s narratives, in the line with previous findings by Cohn and coworkers [18]. Our participants reported either a relief to obtain “a genetic explanation” for VTE episodes, or a distress about transmission to children; others, finally, felt confused by the occurrence of VTE in those family members tested negative for the genes. Most importantly, the concept of unknown blood abnormalities, transmitted within a family and at present undetectable by genetic testing, was apparent in our interviews; as a consequence, all participants did not perceived a negative testing as reassuring.

### Construction of Secondary Prevention Attitudes

Our study offers the opportunity to appraise VTE preventive measures mentioned in each patient or family member narrative. Those preventive measures depend on the perception of the episode context shared within families, whether or not participants reported communication on that topic. Treatment, consisting at that time in anti-vitamin K therapy and leg stockings, was reported as bothersome [19]. Narrative’s analyses showed that emphasis was placed on other preventive measures, broadly promoted for primary and secondary prevention of arterial diseases, such as healthy diet and taking exercise. Underlying concepts on blood circulation and its improvement by those measures were the main pathophysiology explanations present in narratives. From the practitioners’ point of view, eating healthy foods and taking exercise may appear more as signs of appropriation of public health messages rather than as specific preventive action in the field of VTE. Whether, in some patients, adoption of these measures excuses themselves of major preventive VTE measures was not apparent from narratives and deserves future investigation.

Remains of VTE episodes, either symptoms for some patients, or knowledge of sequels for others, were mentioned as reminders of past VTE event, then of possible VTE event recurrence and as a support for secondary prevention compliance [20]. Contrasting with the well-known Health Belief Model, remains and not beliefs are thus presented as determinants of compliance [21, 22]. It is important to note that patients and family members develop autonomous preventive measures that may or may not fit with guidelines and/or physician’s recommendations. Direct information from patient’s relatives point of view on VTE usually lack in daily practice; it is worth however for the practitioner to recall that interpretation of VTE risk and its consequences are shared within the family.

Among symptoms mentioned in narratives, fatigue takes place as a VTE remain as well as a possible risk factor, both resulting in preventive attitudes. Fatigue has previously been assessed by means of a questionnaire in a cohort of more than two hundred patients with a previous VTE and no underlying conditions, such as cancer, chronic illness or immobilization [14]: after controlling for demographic and medical factors, fatigue was negatively associated with quality of life, as measured by SF 12 questionnaire. The authors suggested that since quality of life is a broad concept, disentangling its different components with respect to a variable such a fatigue might provide novel information in terms of tailored interventions. Findings from our study also suggest that it might be useful to elicit with patients whether fatigue is perceived as a consequence and/or a possible cause of VTE. Preliminary studies suggested feasibility of a rehabilitation program after a VTE episode [23]. Whether such programs have the potential to decrease fatigue, as is the case in patients with coronary artery disease, need to be studied prospectively [24, 25].

### Strengths and Weaknesses

This is to our knowledge the first qualitative study involving VTE patients and their relatives. The study design allowed us to see whether patients and first-degree relatives’ relationships influenced acceptance or resistance to adopting preventive measures. The use of narratives took into account interactions with others, as well as personal biography and past history of participants [10]. This qualitative approach allowed us to explore dimensions that were not yet covered by the literature.

Limitations of the study include the fact that patients were participants of a previous study, held in our tertiary hospital, of factors that predict risk of venous thrombosis in first-degree relatives of patients with unprovoked VTE. Patients and family members had thus received results of genetic testing prior to the study. Interviews were conducted mainly in the same location—the outpatient clinic—as for the previous study. Although the interviewer was introduced as an anthropologist, that location might prone patients to focus on medical concerns on the one hand; on the other hand, the interviewer might have benefit from participants trust in a familiar medical team. Despite the fact that participants had prior to the interviews received some degree of information, data from their narratives show reinterpretations of VTE episodes and their further preventive measures. Patients seeking care in other facilities such as primary hospitals might face different problems and interact with a less specialized health care team. For these reasons, our results may not be applicable outside thromboembolic units. [26].

## Conclusion

Our study shows that interpretation of VTE episode and further elaboration of secondary prevention attitudes by patients and their family follow a complex process, only partially influenced by the information given with health care providers. Interactions with health care providers have to been conducted in order to co-construct the meaning of the event. Physical and psychological impact of VTE is associated in some patients with profound biographical disruption. In that instance, a medical team is faced with the challenge to help patient and family members to appraise unprovoked VTE event and potential recurrence, without hampering long-term quality of life. In an attempt to answer these problems, we have now implemented in our clinic a support service that consists in a dedicated appointment, taking place one month after the VTE episode and managed by a team of physicians and nurses. As a first step in an education process, patients [and a family member on patient’s request] are encouraged to provide feedback on the event and its consequences, including daily life and work, information is shared and follow-up scheduled. Pending on the results of this appointment, special needs, such as psychological or social support, are then considered.
